# UAV Based Relay for Wireless Sensor Networks in 5G Systems

**DOI:** 10.3390/s18082413

**Published:** 2018-07-25

**Authors:** Shu Fu, Lian Zhao, Zhou Su, Xin Jian

**Affiliations:** 1 The College of Communication Engineering, Chongqing University, Chongqing 400044, China; jianxin@cqu.edu.cn; 2 The Department of Electrical and Computer Engineering, Ryerson University, Toronto, ON M5B 2K3, Canada; lzhao@ee.ryerson.ca; 3 The School of Mechatronic Engineering and Automation, Shanghai University, Shanghai 200444, China; zhousu@ieee.org

**Keywords:** wireless sensor networks, unmanned aerial vehicle, relay, power consumption

## Abstract

Relay is one of the most significant issues in smart industrial wireless sensor networks (WSN) due to the low transmitting power of sensors. By relay, the signals of sensors can be concentrated at the relay and further transmitted to the base station for decreasing energy consumption in the system. In the past decades, the relay in WSN is generally one super sensor with large transmitting power. However, the placement of the super sensor is static, which leads to the instability of performance in WSN under the time-varying wireless environment. Fortunately, unmanned aerial vehicles (UAV) can provide an effective leverage to improve the environment-adaptation in WSN compared to the static relay in WSN. In this paper, we employ UAV as the relay in WSN, which can move in three-dimensional space to possess a better position to minimize the system power consumption. We use a simple case study to demonstrate the effectiveness of UAV in WSN. Extended simulations are also given to verify the preferable performance of the UAV based relay in WSN.

## 1. Introduction

Smart industrial wireless sensor networks (WSN) [1,2,3] have been widely laid out to meet the ever-increasing demands of the next generation of wireless communication. Smart industrial wireless sensor networks can provide real-time service of smartphones, road condition information for vehicle networks, video-game consoles, surveillance cameras, augmented reality devices and wearable electronics, etc. [4], which can effectively support the implementation of an Internet of Things (IoT) based on the fifth generation of the wireless network (5G) [5]. Most types of sensors possess simplification features and low transmitting power to cut down on the investment cost in practice. However, when the sensors transmit signals to the base station (BS) over a long distance, the low power of the sensors may lead to a poor received signal to interference plus noise ratio (SINR) at the BS side. Relay [6,7,8,9,10] is a useful technology to receive and retransmit the signals of sensors to the BS, where the relay can enhance the strength of signals received at the BS side. In reference [5], the authors pointed out that a relay can achieve anti-fading capability in WSN. In reference [6,7,8,9], power splitting is investigated in a relay. In reference [10], relay-based routing protocol techniques are considered.

Most of the existing works regarding a relay in WSN employ a static or semi-static relay to retransmit the signal of the sensors to the BS. This lacks flexibility of the relay position to better adapt to the time-varying wireless environment. Unmanned aerial vehicles (UAV) [11,12,13,14,15] provide a useful leverage when they act as the relay in WSN; they can move in the three-dimensional space for better adaptation to the wireless environment. Data gathering via UAV in WSN has been widely studied in the existing works [16,17]. However, the optimal staying position of UAV remains an open problem.

By the observations above, we are motived to combine sensors and UAV as an integration, where sensors first transmit signals to a concentrator. Then, the concentrator data will be retransmitted and relayed by UAV to the BS side. By the transmitting power of the UAV, the received SINR of the signals at the BS side can be largely enhanced. Through this UAV based relay mechanism in WSN, the transmitting power of the concentrator can be cut down to meet the practical configuration of WSN. On the other hand, the specific staying position of the UAV based relay in the three-dimensional space should be appropriately determined to achieve the tradeoff of transmitting power between the concentrator and UAV, which can minimize the system’s energy consumption. In this paper, in order to determine the optimal staying position of the UAV based relay, we first propose the architecture of the UAV based relay in WSN with the channel model using the fifth generation of wireless communication (5G). Then, we propose a Location of UAV (LU) algorithm based on the channel gain to obtain the optimal staying position of the UAV, which can be dynamically adjusted according to the changing wireless channel gain.

The remainder of the paper is organized as follows: Section 2 provides the system model and the optimization problem model. Section 3 proposes the algorithm to determine the position of UAV. Section 4 further verifies the effectiveness of our proposed algorithm via extended simulations. In Section 5, we conclude this paper.

## 2. System Model and the Problem Formulation

**Figure 1:**
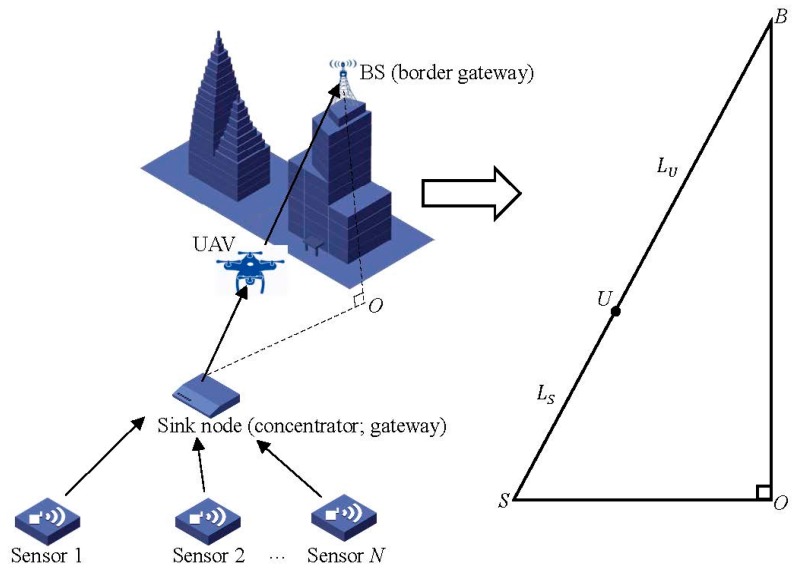
System model of the unmanned aerial vehicle (UAV) based relay in wireless sensor networks (WSN).

In this section, we introduce the system model of the UAV based relay in WSN. As shown in Figure 1, users contain different types of sensors (sensors, road side units, etc.), and the data sensed by each one will be collected by a concentrator (sink node/gateway). Moreover, the concentrator will retransmit the collected data to the BS, which connects the WSN to the Internet. Hence, the concentrator acts as a gateway to concentrate the traffic data sensed in WSN for transmission to the border gateway (BS). Then, the WSN can connect to the Internet.

Notably, in this paper, we assume that WSN can work at 900 MHz/2.4 GHz to support the 5 GHz frequency bands, and the concentrator contains two interfaces in order to connect WSN (Zigbee, WiFi, 6LoWPAN, etc.) to the 5G mobile communications world. The approach we propose to improve the system performance is employing the UAV as a relay station, where we consider the path among SINK-UAV-BS to simplify the system model. Then, the omnidirectional path model can thus be used in this paper.

We assume the wireless bandwidth of the concentrator at the sink node is $B_{S}$ Hz, and the wireless bandwidth of the UAV is $B_{U}$ Hz. The transmitting power of the concentrator is denoted as $P_{S}$, and the transmitting power of the UAV is denoted as $P_{U}$. The power of Gaussian White Noise is denoted as $\sigma^{2}$. The wireless channel gain from the concentrator to the UAV is denoted as ${\left|h_{S}\right|}^{2}$, and the wireless channel gain from the UAV to BS is denoted as ${\left|h_{U}\right|}^{2}$. In this paper, the omnidirectional path model [18] based on the millimeter waves (mmWave) model is employed as in (1). However, the algorithm and results in this paper can also apply to the existing scenarios of WSN

(1) $$L=86.6+24.5\times \log_{10}d\;\left(\mathrm{dB}\right),$$

where *L* denotes path loss in decibels (dB), and *d* denotes the distance between the source and end in meter. To make the paper more focused, we ignore the impact of fast fading and slow fading on the wireless channel gain. We make such an assumption from the following two reasons: first, the impact of fading can be counteracted from the long-term average. Second, we can obtain a more theoretical analysis to guide the engineering.

Define $L_{S}$ as the distance between the concentrator at the sink node and UAV, and $L_{U}$ as the distance between UAV and BS, respectively. By (1), we can further derive ${\left|h_{S}\right|}^{2}$ and ${\left|h_{U}\right|}^{2}$ as in (2) and (3), respectively.

(2) $${\left|h_{S}\right|}^{2}=10^{-\frac{L_{S}}{10}},$$

(3) $${\left|h_{U}\right|}^{2}=10^{-\frac{L_{U}}{10}}.$$

To simplify the system model, in this paper we assume the concentrator transmit signal to the BS with one UAV as the relay. This system model can better reveal the relationship between the position of the UAV based relay and the system energy consumption. As shown in Figure 1, sensors first transmit signals to the concentrator. Then, the concentrator retransmit the signal to the BS, which will deliver the wireless data to the destination in the network.

The model can be abstracted as the right triangle at the right side in Figure 1. The vertical dimension from BS to the ground is denoted as segment $\bar{BO}$ in meter (m), and the horizontal distance between the concentrator and BS is denoted as $\bar{SO}$ in meter. Then, the sight distance between the concentrator and BS is

(3) $$\bar{BS}=\sqrt{{\bar{BO}}^{2}+{\bar{SO}}^{2}}\;\left(m\right).$$

**Figure 2:**
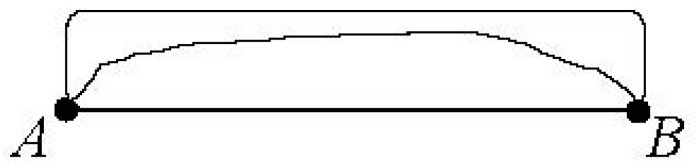
Distance between two points (The line segment between two points is the shortest).

The optimal position of the UAV will be dotted at the segment $\bar{BS}$ because the minimize distance between two arbitrary points is the line segment. As shown in Figure 2, the minimized total distance between point *A* and *B* is the line segment $\bar{AB}$. The minimized transmission distance between BS and the concentrator can minimize the transmitting power of the concentrator and UAV because the path loss is in direct proportion to the transmitting distance according to (1).

The key issue is to determine the specific position of the UAV at the line segment $\bar{BS}$. We define the length of the line segment $\bar{US}$ as *x*, the length of $\bar{BS}$ is *T*, then the length of $\bar{BU}$ is T−x. Assume that the demand of traffic capacity is *C* bit/s (bps) for transmission of the concentrator. To avoid packet loss at the UAV, the capacity for transmission of UAV is also *C* bps. Let $\sigma_{S}$ and $\sigma_{U}$ denote the noise power received at UAV and BS, respectively. According to Shannon capacity, we have

(4) $$C=B_{S}\;\log_{2}\left(1+\frac{P_{S}{\left|h_{S}\right|}^{2}}{\sigma_{S}{}^{2}}\right)=B_{U}\;\log_{2}(1+\frac{P_{U}{\left|h_{U}\right|}^{2}}{\sigma_{U}{}^{2}}),$$

(5) $${\left|h_{S}\right|}^{2}=10^{-\frac{L_{S}}{10}}=10^{-\frac{86.6+24.5\times \log_{10}x}{10}},$$

(6) $${\left|h_{U}\right|}^{2}=10^{-\frac{L_{U}}{10}}=10^{-\frac{86.6+24.5\times \log_{10}\left(T-x\right)}{10}}.$$

By (4), we can further prove the transmitting power of the concentrator and UAV as

(7) $$P_{S}=\frac{\sigma_{S}{}^{2}}{{\left|h_{S}\right|}^{2}}\left(2^{\frac{C}{B_{S}}}-1\right)=\frac{\sigma_{S}{}^{2}}{10^{-\frac{86.6+24.5\times \log_{10}x}{10}}}\left(2^{\frac{C}{B_{S}}}-1\right),$$

(8) $$P_{U}=\frac{\sigma_{U}{}^{2}}{{\left|h_{U}\right|}^{2}}\left(2^{\frac{C}{B_{U}}}-1\right)=\frac{\sigma_{U}{}^{2}}{10^{-\frac{86.6+24.5\times \log_{10}\left(T-x\right)}{10}}}\left(2^{\frac{C}{B_{U}}}-1\right).$$

The objective is to minimize the system transmitting power in WSN. We denote the total transmitting power in the system as $P_{\max}$, then, we can obtain the system optimization model as

(9) $$\mathrm{minimize}\;P_{\max}=P_{S}+P_{U}=\frac{\sigma_{S}{}^{2}}{10^{-\frac{86.6+24.5\times \log_{10}x}{10}}}\left(2^{\frac{C}{B_{S}}}-1\right)+\frac{\sigma_{U}{}^{2}}{10^{-\frac{86.6+24.5\times \log_{10}\left(T-x\right)}{10}}}\left(2^{\frac{C}{B_{U}}}-1\right).$$

## 3. Convex Optimization Based Position of Unmanned Aerial Vehicle

To obtain *x* for minimizing $P_{\max}$ in (9), convex optimization can be employed. We can first simplify (9) as

(10) $$\mathrm{minimize}\;\varphi =\sigma_{S}{}^{2}\left(2^{\frac{C}{B_{S}}}-1\right)x^{2.45}+\sigma_{U}{}^{2}\left(2^{\frac{C}{B_{U}}}-1\right){\left(T-x\right)}^{2.45}.$$

By deriving the second order partial derivative of φ, we can have $\varphi^{\prime \prime }>0$. This suggests that φ is a convex function with *x*, and thus the minimal φ exists. By the first order partial derivative of φ, we can obtain the optimal *x* for minimizing $P_{\max}$.

(11) $$x=\frac{T}{{\left(\frac{W_{1}}{W_{2}}\right)}^{\frac{1}{1.45}}+1},$$

where $W_{1}=\sigma_{S}{}^{2}(2^{\frac{C}{B_{S}}}-1)$, and $W_{2}=\sigma_{U}{}^{2}\left(2^{\frac{C}{B_{U}}}-1\right)$. By (11), we can obtain $L_{S}=\frac{T}{{\left(\frac{W_{1}}{W_{2}}\right)}^{\frac{1}{1.45}}+1}$, and $L_{U}=T-x$. We can then propose the location of UAV (LU) algorithm (Algorithm 1) to determine the best position of UAV as a relay in WSN. Specifically, we first calculate the input parameters in the LU algorithm, then, $\bar{BS}$ will be obtained by (3). Considering the optimal objective in (9), by the discussion above, we can obtain the optimal staying position of UAV; i.e., the optimal *x* in (11).

**Algorithm 1.** Location of UAV (LU) algorithm.**Input**: location of the concentrator, location of BS, the length of $\bar{BO}$ and $\bar{SO}$, respectively; *C*, $B_{S}$, $B_{U}$, $P_{S}$, $\sigma_{S}{}^{2}$, $\sigma_{U}{}^{2}$ and $P_{U}$.**Output**: the optimal $L_{S}$ for minimizing system power consumption.**1.** Determine $\bar{BS}$ by (3). **2.** Obtain the optimization model in (9). **3.** Obtain the optimal value of $L_{S}$ as in (11). **Return:**
$L_{S}$.

## 4. Simulation

In this section, we explore the performance of the LU algorithm in UAV based relay in WSN in the 5G scenario with the corresponding parameters in Table 1. Unless otherwise specified, the parameters are defined in Table 1. In this paper, we compare the performance of LU with the traditional wireless transmission without relay; i.e., direct transmission. The simulation is based on Matlab 2013b.

**Figure 3:**
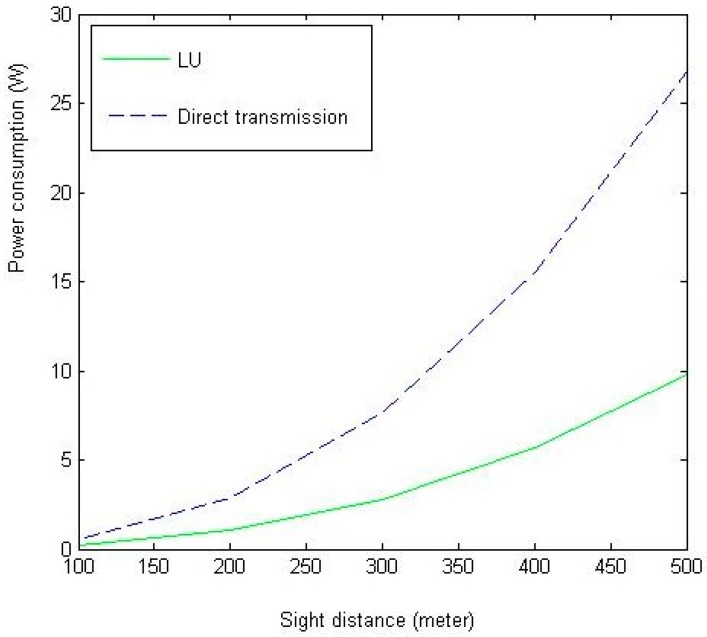
The impact of *T* on system power consumption (the amount of total power needed in system under different configurations of sight distance).

In Figure 3, we show the relationship between sight distance *T* and the system power consumption $P_{\max}$. As shown in Figure 3, the LU algorithm can largely cut down the power consumption due to the relay via UAV. As *T* increases, the gap between the two algorithms enlarges, which suggests that the effectiveness of the UAV based relay in WSN.

**Figure 4:**
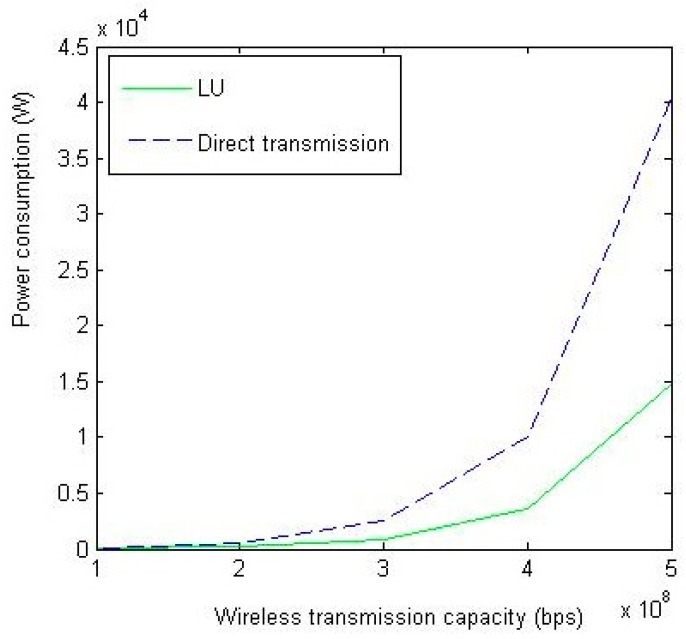
The impact of *C* on system power consumption (the total power consumed in system under different configurations of wireless transmission capacity (bps)).

In Figure 4, we study the impact of *C* on system power consumption. Obviously, as *C* increases, the power consumption increases due to the Shannon capacity. The performance of LU still outperforms the direct transmission method.

**Figure 5:**
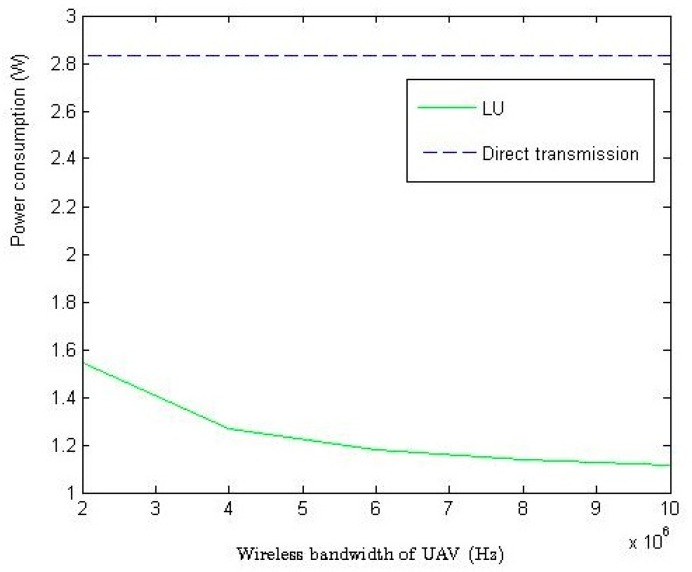
The impact of $B_{U}$ on system power consumption (how to cut down the power consumed in system by increasing wireless bandwidth of UAV (Hz)).

In Figure 5, we study the impact of $B_{U}$ on the system power consumption. Since direct transmission method does not have a UAV, the power consumption remains. As shown in Figure 5, LU algorithm can largely cut down the power consumption as $B_{U}$ increases, because the increased wireless bandwidth of UAV can decrease the demanded power for transmission in WSN based on Shannon capacity.

**Figure 6:**
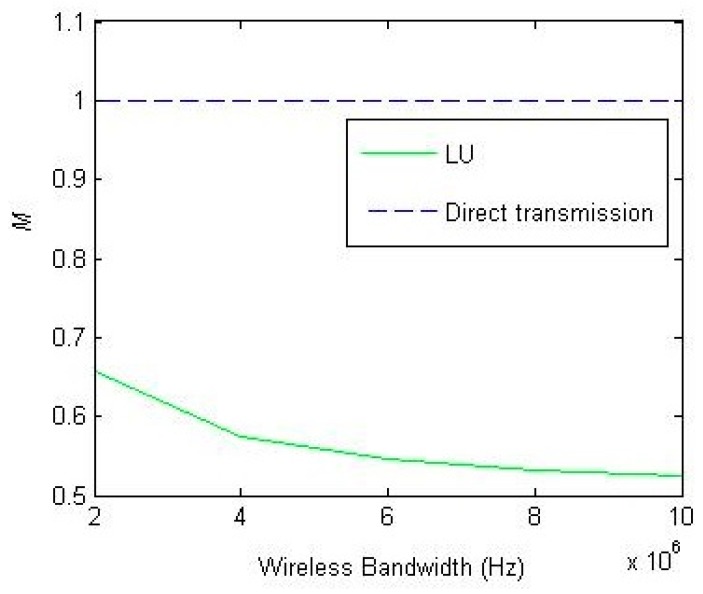
The impact of $B_{U}$ on $M=\frac{x}{T}$ (how to place the UAV under different configurations of $B_{U}$).

In Figure 6, we define $M=\frac{x}{T}$ as the proportion of UAV position to *T*. As Figure 6 shows, as $B_{U}$ increases, the UAV will move towards the concentrator. This can cut down the power of the concentrator, meanwhile the increase of UAV power can be controlled due to the increased wireless bandwidth of UAV.

## 5. Research Direction and Conclusions

**Figure 7:**
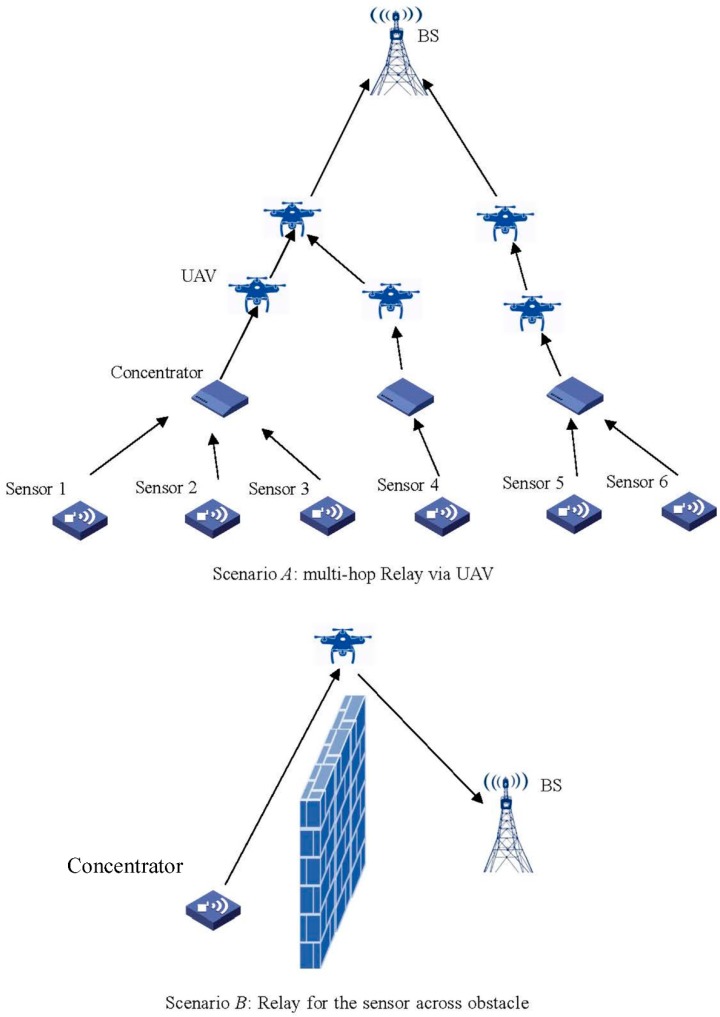
Scenarios of UAV based relay in WSN.

We have introduced the LU algorithm to determine the position of a UAV in WSN. However, to simplify the paper, we use an oversimplified model to reveal the relationship between the three-dimensional positioning of the UAV and the system energy consumption, where the three-dimensional space has been considered in only one dimension. In the future, we should further extend the work into more practical scenarios. In Figure 7, we give several scenarios based on the UAV in WSN. For the Scenario *A* in Figure 7, when multiple concentrators and UAV exist in system, the position of the UAV will largely affect the performance of WSN. Unlike the case in Figure 2, when multiple UAVs are considered the optimal position of the UAV cannot be directly obtained by the LU algorithm. To network the relays in WSN and determine the number of relay stages in WSN will be our future work. At the bottom of Figure 7, we map out the second scenario for a UAV based relay. In this case, to avoid the obstacles blocking the wireless transmission in sight distance, the UAV can act as the relay between the source and the destination for the signal transmitting.

**Figure 8:**
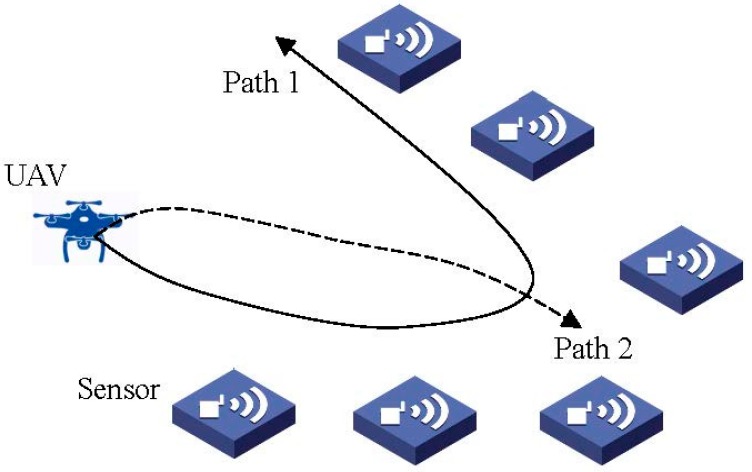
Flight path selection of UAV.

On the other hand, the flightpath selection algorithm of UAV will be proposed in our future work to achieve the best path for data collection. As shown in Figure 8, different paths of UAV will determine the different wireless channel gains employed to collect the concentrator data. Unlike the model in this paper, UAV in Figure 8 can fly in three-dimensional space. The optimal flightpath can be determined by machine learning, etc., which will be left for our future work.

Moreover, several issues should be further studied in future work to increase practicality. We observe the issues as follows:

(1) When frequency subchannels, fast fading, and slow fading are considered, the optimal position for a specific UAV differs across the frequency of subchannels. How to determine the optimal position will be an intractable problem.
(2) Considering the power consumption of UAV flight, how does the optimal position of a UAV change.
(3) How to determine the optimal flightpath. This is a key issue for the UAV based relay due to the moving feature of a UAV.
(4) How to achieve the tradeoff between the number of UAV and the performance of the WSN. This should be appropriately determined by an optimization tool, such as geometric programming optimization, etc.
(5) A caching [19,20,21] based UAV relay will be investigated where queuing theory should be employed to analyze the performance of the WSN system.

In conclusion, this paper aims to propose the UAV based relay in WSN to determine the optimal position of UAV based on the wireless environment for minimizing the system’s power consumption. Such an architecture can effectively make up for the disadvantage of the small transmitting power of the wireless devices in WSN. As $B_{U}$ increases, a UAV can approach the concentrator (i.e., *M* decreases) to decrease the transmitting power of the concentrator. To determine the optimal staying position of the UAV, we have also proposed a simple scenario and the corresponding LU algorithm based on the revealed philosophy of the UAV position to obtain the optimal position minimizing the system power consumption. We have verified the effectiveness of the proposed algorithms via extended simulations.

## Figures and Tables

**Table 1:** Simulation Parameter for Location of UAV (LU) algorithm.

$B_{S}$	50 MHz
$B_{U}$	50 MHz
*T*	200 m
*C*	500 Mbps
Noise power spectral density	−174 dBm/Hz
The omnidirectional path model	$86.6+24.5\;\log_{10}\;d$
Link	Uplink
